# Effect of Co-Doping of Al^3+^, In^3+^, and Y^3+^ on the Electrical Properties of Zinc Oxide Varistors under Pre-Synthesizing BiSbO_4_

**DOI:** 10.3390/ma17061401

**Published:** 2024-03-19

**Authors:** Bo Xu, Lei Wang, Mengfan Yang, Yu Xiang, Lingyun Liu

**Affiliations:** 1 School of Science, Hubei University of Technology, Wuhan 430068, China; 102112278@hbut.edu.cn (B.X.); 102112279@hbut.edu.cn (L.W.); 102112292@hbut.edu.cn (M.Y.); 102202256@hbut.edu.cn (Y.X.); 2 Hubei Collaborative Innovation Center for Efficient Utilization of Solar Energy, Hubei University of Technology, Wuhan 430068, China

**Keywords:** zinc oxide varistors, pre-synthesis, doping, electrical properties

## Abstract

Under the premise of using the solid-phase method to pre-sinter Bi_2_O_3_ and Sb_2_O_3_ into BiSbO_4_ as a substitute for equal amounts of Bi_2_O_3_ and Sb_2_O_3_ in the formula, the effects of co-doping with In(NO_3_)_3_, Al(NO_3_)_3_, and Y(NO_3_)_3_ on the microstructure and electrical properties of ZnO varistors were studied. The experimental results show that with an increase in In^3+^-doped molar concentration, the leakage current of the ZnO varistor shows a rapid decrease and then a slow increase trend. However, the nonlinear coefficient is the opposite of it. With the combined effect of the rare earth element Y^3+^, the average grain size is significantly reduced, which leads to an increase in the voltage gradient. At the same time, a certain amount of doped In^3+^ and Al^3+^ is dissolved into the grains, resulting in a decrease in grain resistance and thus a low level of residual voltage. The varistor with 0.6 mol% In^3+^, 0.1 mol% Al^3+^, and 0.9 mol% Y^3+^ doping ratios exhibits excellent overall performance. The nonlinear coefficient is 62.2, with the leakage current being 1.46 µA/cm^2^ and the voltage gradient being 558 V/mm, and the residual voltage ratio is 1.73. The prepared co-doped ZnO varistors will provide better protection for metal oxide surge arresters.

## 1. Introduction

Zinc oxide varistor is a kind of polycrystalline semiconductor ceramic with a complex, multi-component oxide composition. The main component is zinc oxide, accounting for more than 90% (molar percentage), with a small amount of Bi_2_O_3_, Sb_2_O_3_, MnO_2_, Cr_2_O_3_, SiO_2_, Co_2_O_3_ and other auxiliary components [1,2,3,4]. In the 1970s, ZnO-Bi_2_O_3_ ceramics were invented by Matsuoka et al. ZnO varistors are widely used as the core components of high-voltage surge arresters and low-voltage surge protection devices because of their excellent nonlinear electric field-current density relationship and their ability to absorb energy surges [1,3]. In the Bi_2_O_3_-ZnO varistor, Bi_2_O_3_ is the main reason for the nonlinear characteristics of the varistor [5]. Bi_2_O_3_ has a low melting point [6], and during the sintering cooling process. Bi_2_O_3_ [7,8] can form a liquid phase, make the dopant more uniformly dispersed, and promote the material exchange between grains and grain boundaries, so that more cations of unsaturated excess metal oxides, such as Co^3+^ and Mn^4+^, can obtain electrons and transform from the high valence state to the stable low valence state. Oxides such as Co_2_O_3_ and MnO_2_ [4,9,10] have host-accepted properties, and their reaction at grain boundaries increases the surface density of states (*N_s_*) of ZnO grains, which leads to a significant increase in the height of the grain boundary layer potential barrier, and thus improves the voltage gradient (E_1mA_) of the sample. At the same time, symmetric double Schottky barriers ($\emptyset_{b}$) are generated at the grain boundaries, and the higher the $\emptyset_{b}$ are the larger the nonlinear coefficients (α). The oxygen desorption mechanism [11,12,13] is used to explain the merits of Bi-rich phases during high temperature sintering. The adsorbed oxygen [14] can introduce interface acceptor states and capture negative charge carriers, thus increasing the double Schottky barrier ($\emptyset_{b}$) in the grain boundary. This is because the interface acceptor state and the donor concentration together determine the height of ${\;\emptyset }_{b}$. However, the low melting point during the high-temperature sintering can also leads to its volatiliztion, and some pore can be formed inside the varistor, which affects its electrical properties [14]. According to the theory of charge compensation mechanism [15], oxygen vacancies, the main transportation channel of adsorbed oxygen, will be consumed in the solid solution process between additives with Bi_2_O_3_, which cause $\emptyset_{b}$ to weaken. Accordingly, BiSbO_4_ can be pre-synthesized using the materials Bi_2_O_3_ and Sb_2_O_3_ instead of the equal amounts of Bi_2_O_3_ and Sb_2_O_3_ in the original formulation. The depletion of oxygen vacancies at grain boundaries during sintering can be avoided increasing the concentration of adsorbed oxygen, and the densification and electrical properties of varistor can be improved [16]. With the increasing application of high-voltage transmission, high-voltage transmission systems are increasingly pursuing smaller and lighter surge arresters, which can effectively improve the uneven potential distribution along the ZnO varistor columns within the arrester [17]. At the same time, this puts higher requirements on the voltage gradient of the piezoresistors. It has been shown that decreasing the sintering temperature and holding time improves the voltage gradient but leads to a decrease in energy absorption capacity and nonlinearity [18,19]. Increasing the voltage gradient by sacrificing other electrical properties is not worth. The increasing the voltage gradient is equivalent to reducing the grain size, and it may be more effective to dope with additives that can inhibit grain growth and obtain a high voltage gradient of the varistors.

Therefore, it is particularly important to improve voltage gradients and nonlinear coefficients while taking into account other electrical characteristics. Al^3+^ doping leads to lower grain resistance and a smaller residual voltage [20,21]. The smaller the grain resistance, the smaller the residual voltage ratio, thus improving the protection performance of the arrester [22]. Yet, it leads to a significant increase in leakage current, shortening the service life of the arrester, and the voltage gradient is somewhat affected [20,23]. Some studies have shown that the doping of In^3+^ can effectively suppress the leakage current, improve the nonlinear coefficient, and significantly reduce the residual voltage ratio, but the doping of In^3+^ does not significantly improve the voltage gradient [9,22,24]. Also, it has been shown that BiSbO_4_ promotes grain growth, which is detrimental to obtaining ZnO varistors with high voltage gradients.The addition of rare-earth oxides [25,26] can effectively improve the grain boundary characteristics and thus enhance the electrical performance of ZnO pre-synthesizing ceramic materials. The rare earth element Y^3+^ forms a bismuth-rich second phase containing Y^3+^ in the sintered composition, which is mainly distributed at the junctions of the three grains and plays the role of “pinning” [27,28], similar to the inhibition effect of spinel on the relative grains, reducing the grain size and significantly improving the potential gradient. It also increases the number of grain boundaries, enlarges the number of Schottky barriers, and improves the nonlinear coefficients to some extent [27,29,30]. This precisely compensates for the disadvantages of ln^3+^ doping, which only slightly increases the voltage gradient of ZnO varistors, and BiSbO_4_ doping, which promotes grain growth.

In order to prepare and obtain varistors with a high voltage gradient, a low residual voltage ratio, and a low leakage current. Therefore, experiments with different doping concentrations of In^3+^ were carried out to investigate the effect of combined doping of the three on the microstructure and electrical performance of the oxide varistor at given Al^3+^ and Y^3+^ doping concentrations and pre-synthesizing BiSbO_4_ in lieu of doping.

## 2. Materials and Methods

**Figure 1:**
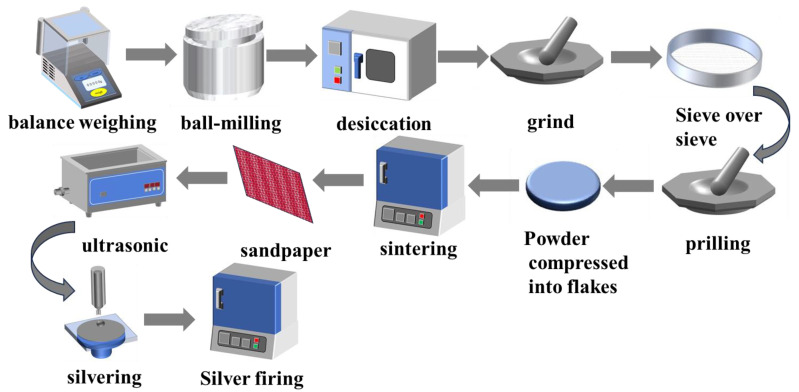
Detailed preparation flow of a varistor.

Co-doped ZnO varistors were prepared from the following compositions: (93.9-X) mol% ZnO, 1 mol% Co_2_O_3_, 0.8 mol% MnO_2_, 1.2 mol% SiO_2_, 2 mol% BiSbO_4_, 0.2 mol% Al(NO_3_)_3_·9H_2_O, 0.9 mol% Y(NO_3_)_3_·6H_2_O, X mol% In (NO_3_)_3_ (X = 0, 0.25, 0.5, 0.75, 1.00). All of the above experimental materials were analytically pure (Shanghai McLean Biochemical Technology Co., Ltd., Shanghai, China). Bi_2_O_3_ and Sb_2_O_3_ were accurately weighed in a molar ratio of 1:1, ball-milled (SFM-1 planetary ball mill, Hefei Kejing Materials Technology Co., Ltd., Hefei, China) for 12 h, dried, heat-treated at 800 °C for 2 h, and naturally cooled to 500 °C for 2 h of annealing treatment to obtain BiSbO_4_. The main raw materials, zinc oxide, pre-synthesizing BiSbO_4_, and its additives were then accurately weighed in molar proportions into the ball-milling jars and then mixed with the appropriate proportion of deionized water and grinding media (zirconia beads). The powder, grinding media, and surfactant (ammonium polyacrylate) were mixed with a suitable proportion of deionized water and then ball milled in a planetary ball mill with a combination of forward and reverse rotation at a speed of 450 r/min for 12 h. The slurry was removed and dried in a blast oven at 110 °C for 12 h. The dried powder was crushed, and a 5 wt% solution of polyvinyl alcohol (PVA) binder was added at a powder-to-PVA mass ratio of 1:9. The powder was ground in an onyx grinding bowl and passed through an 80 mesh stainless steel sieve for granulation to obtain the starting powder. 1 g of powder is weighed and pressed uniaxially at 10 Mpa for 90 s to form discs of 15 mm diameter and 1 mm thickness. Sintering was carried out in an air environment by placing the pressed discs in a muffle furnace (KSL-1700X, Hefei Kejing Materials Technology Co., Ltd., Hefei, China) and holding them at 400 °C for 4 h to release the binder (PVA), then increasing the temperature to 1100 °C and holding for 3 h. It is important to note that the temperature interval from 800 °C to 1000 °C during warming is the joint stage of densification of the varistors, while the temperature interval from 950 °C to 800 °C during cooling is critical for the nonlinear coefficient. In these two specific temperature intervals, the rate of temperature rise or fall should be slowed down to 2 °C/min and 1 °C/min, respectively. After the temperature was reduced to room temperature, the upper and lower surfaces of the ZnO varistors were sanded and polished, ultrasonically cleaned and dried, and then silver paste was applied to the upper and lower surfaces of the discs. 500 °C holding time of 60 min was used for curing to form the ohmic contacts as test electrodes. The flow chart for the preparation of ZnO varistors is shown in Figure 1.

In addition, the microstructure of the cross-section of the sintered samples was observed using an electron microscope (SEM, JSM-6380, Japan Electronics Co., Ltd., Tokyo, Japan). The average grain size (d) was determined by the formula of the linear intercept method: d = 1.56 L⁄MN, where L is the length of a randomly delineated measurement line on the SEM image, M is the magnification of the SEM image, and N is the number of ZnO varistor boundaries covered by the delineated measurement line. X-ray diffraction (XRD) maps of the samples were obtained using CuKa radiation (XRD-7000X, Shimadzu Corporation, Kyoto, Japan) to determine the grain boundary structure and phase composition of the piezoresistors. The samples were scanned under CuKa radiation at a wavelength of 0.1540 nm at a scanning rate of 5° per minute over a diffraction angle range of 10° to 80°.The electric field-current density (E-J) relationship curves in the pre-breakdown and breakdown zones were measured using a source meter (Keithley 2410, Keithley Instruments, Inc., Cleveland, OH, USA) to calculate the electrical characteristics of the piezoresistors. For a given current density of 1.0 mA/cm^−2^, the voltage drop across the piezoresistor is the breakdown voltage (U_1mA_). The relationship between the voltage gradient (E_1mA_) and U_1mA_: E_1mA_ = U_1mA_/D (D is the thickness of the varistor). The leakage current density (IL) was tested at 0.75E_1mA_ electric field strength. The nonlinear coefficient was calculated as: $\alpha =(logI_{2}-logI_{1})/\left(logV_{2}-logV_{1}\right)$, where *V_2_* and *V_1_* are the electric fields at current densities of 1.0 mA/cm^2^ and 0.1 mA/cm^2^, respectively. The capacitance-voltage (C-V) characteristics were measured using a broadband dielectric device (Novocontrol Concept 80, Novocontrol Technologies, Montabaur, Germany) at a frequency of 1 kHz. The residual voltage ratio (K_p_) was calculated as K_P_ = Un/E_1mA_, where Un is the residual voltage at a pulse current density of 63.7 A/cm^2^. A pulse generator (BS1005 8/20 µs) was used to generate a pulse current with a waveform of 8/20 µs. Every eight samples constitute a group, and the data in the text are their mean values to eliminate the effect of chance error on the reliability of the data.

## 3. Results and Discussion

**Figure 2:**
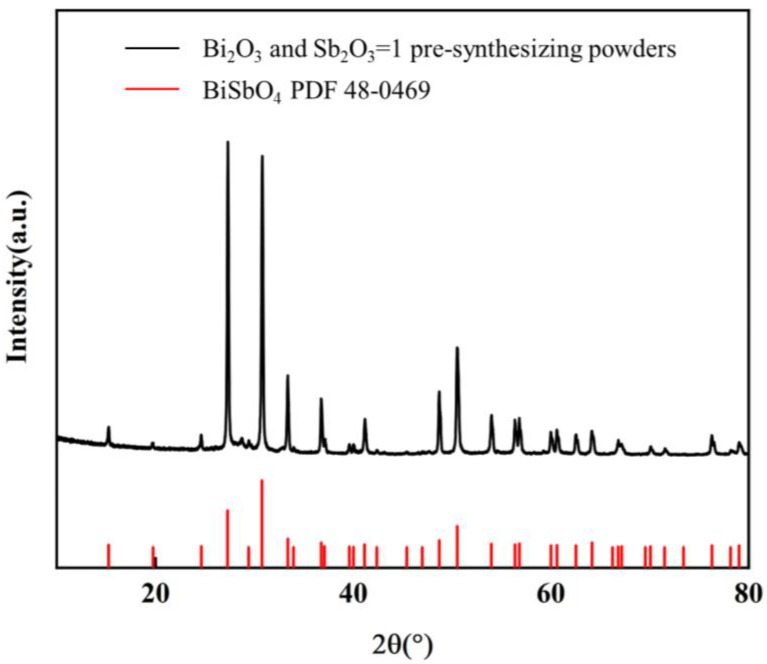
XRD patterns of the pre-synthesized BiSbO_4_ phase.

Figure 2 shows the XRD patterns of the complexes of Bi_2_O_3_ and Sb_2_O_3_ mixed in a 1:1 ratio and sintered at 800 °C for 2 h. The diffraction angles of the three strongest peaks correspond to about 26°, 30°, and 50°, and all the diffraction peaks coincide with the BiSbO_4_ standard card (JCPDS card No. 48-0469). Bi_2_O_3_ and Sb_2_O_3_ diffraction peaks were not detected, and there were no secondary phases. This indicates that Bi_2_O_3_ and Sb_2_O_3_ reacted completely at 800 °C without residual impurities, and the sintered complex is a high-purity BiSbO_4_. The chemical reaction equation is:

(1) $${Bi}_{2}O_{3}+{Sb}_{2}O_{3}+O_{2}⟶BiSbO_{4}$$

**Figure 3:**
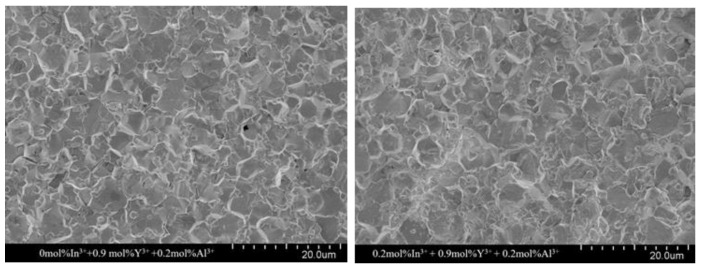

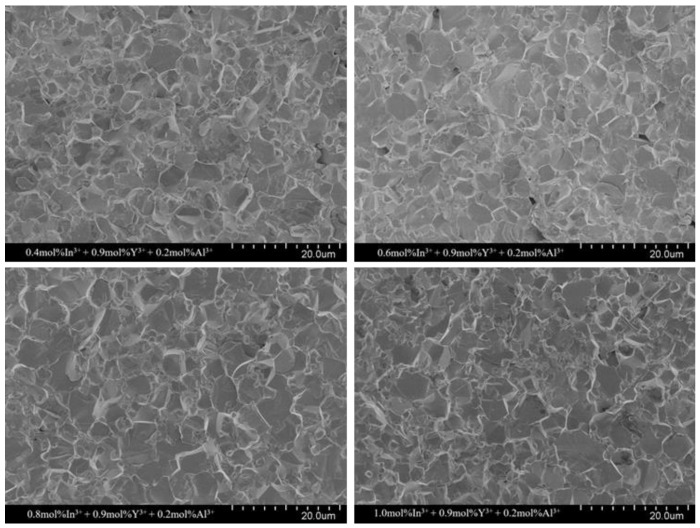
Electron microscope scans (SEM) of different doping ratios of In^3+^.

The microstructure of the sintered ceramics with different In^3+^ doping molar ratios is shown in Figure 3. The average grain size (d) was calculated by the above intercept method and is listed in Table 1. The grain size decreases gradually from 6.31 µm to 4.46 µm with an increasing In^3+^ doping ratio. This can be explained by the fact that some doped In^3+^ form small particles at the grain edges, which act similar to the “pinning” effect of spinel and inhibit grain growth. This is consistent with the effect of added In^3+^ on grain size reported by others [22,24]. It was also observed that at a doping concentration of 0.6 mol% of In^3+^ ions, the homogeneity of the grains was better, which was favorable for the electrical properties of the ZnO resistor sheet [31].

**Figure 4:**
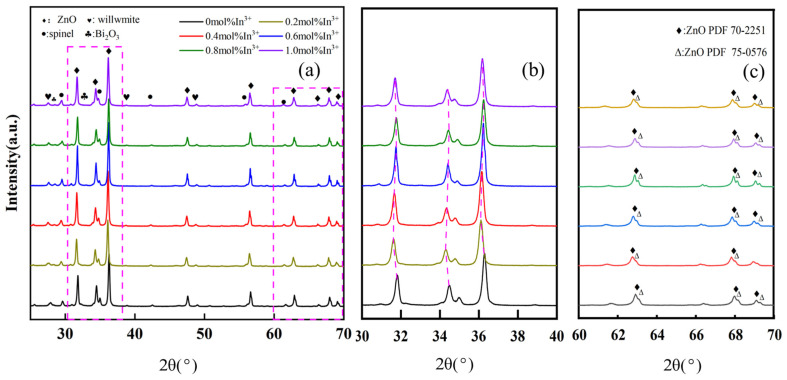
Different In^3^+ doping concentrations (piezoresistor XRD patterns) sintered at 1100 °C for 3 h. (**a**) XRD plots of ZnO varistors with different In^3+^ doping concentrations; (**b**) XRD plots of the three strongest peaks of the ZnO varistor sample; (**c**) Localized magnified XRD plots with diffraction angles between 60° and 70°.

Figure 4 shows the XRD patterns of ZnO varistor ceramics sintered at 1100 °C for 3 h with different amounts of In^3+^ doping, which are predominantly hexagonal-structured ZnO (PDF 70-2251 and PDF 75-0576), and a few amounts of second phases, including spinel, willemite, and Bi-rich phases. The presence of the Bi-rich liquid phase is mainly to provide a place for the dissolution and reaction of other additives to promote the sintering of ceramics and make the reaction more complete. From Figure 4a, it is clearly observed that the diffraction intensity of the strongest peak (diffraction angle of about 38°) and the second strongest peak (diffraction angle of about 32°) is maximum at 0.6 mol% doping concentration of In^3+^ as compared to other doping concentrations. This indicates that the crystallinity of ZnO grains is higher when the doping concentration of In^3+^ is 0.6 mol%.

Notably, it can be observed from Figure 4b that the diffraction angles of the three strongest peaks slightly tend to move to lower diffraction angles with the increase of the In^3+^ doping ratio, and an inflection point occurs at the In^3+^ doping concentration of 0.6%. At 0.6% to 1.0%, the diffraction angles of the three strongest peaks are almost unchanged. The ZnO lattice parameter a = 3.1258 Å and c = 5.2213 Å is used when there is no doping of In^3+^. As the concentration of In^3+^ increases, In^3+^ enters the ZnO lattice and replaces the defects in the position of Zn^2+^, which results in an increase in the lattice parameter. The radius of In^3+^ is larger than that of Zn^2+^, and there is a limit to the solid solution reaction inside the grains. When the doping concentration reaches 0.6 mol%, the lattice parameter tends to be stabilized at a = 3.3591 Å and c = 5.6318 Å. In the doping concentration of In^3+^ from 0.6 mol% to 1.0 mol%, the lattice parameter of ZnO is basically unchanged. This slight decrease in lattice parameter values. This is due to the presence of small stresses in the ZnO structure, which leads to the enlargement of the single cell of the ZnO lattice [32]. This can also be explained by the fact that at low doping concentrations [33], In^3+^ is rapidly and mostly solidified into the ZnO lattice, but the solidification is limited by the fact that the ionic radius of In^3+^ (ion radius of 8.0 × 10^−11^ m) is larger than that of Zn^2+^ (ion radius of 7.4 × 10^−11^ m), and thus the diffraction angles of the three strongest peaks are almost the same at doping ratios of 0.6%, 0.8%, and 1.0%. In the doping concentration of In^3+^ from 0.6 mol% to 1.0 mol%, the lattice parameter of ZnO is basically unchanged. This is due to the presence of small stresses in the ZnO structure, which leads to the enlargement of the single cell of the ZnO lattice. This can also be explained by the fact that at low doping concentrations, In^3+^ is rapidly and mostly solidified into the ZnO lattice, but the solidification is limited by the fact that the ionic radius of In^3+^ (ion radius of 8.0 × 10^−11^ m) is larger than that of Zn^2+^ (ion radius of 7.4 × 10^−11^ m), and thus the diffraction angles of the three strongest peaks are almost the same at doping ratios of 0.6%, 0.8%, and 1.0%. At the same time, this also confirms the theory that “larger ion doping will move the XRD diffraction peaks to a lower diffraction angle, while smaller radius ion doping will move the corresponding diffraction peaks to a higher diffraction angle” [32,34]. This peak shift indirectly reflects the solid solution of In^3+^ ions in ZnO grains. In Figure 4c, the detachment of the ZnO diffraction peaks with lower intensities is observed in the diffraction interval with diffraction angles from 60° to 70°, which again suggests that the doping of In^3+^ distorts the ZnO lattice. There are two main reasons for the absence of diffraction peaks of the corresponding substances of other ions in the XRD spectra: it is related to the ionic radius, and the additive ions with smaller ionic radius are easy to be doped into the ZnO crystal lattice; and some of the additive ions are supposed to be due to the lower concentration of doping, and so the diffraction peaks of the corresponding ions do not appear.

**Figure 5:**
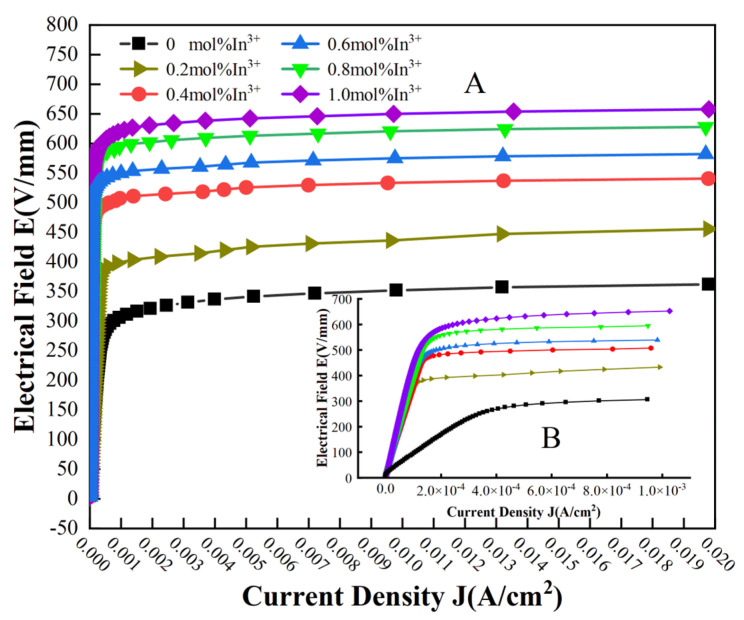
(**A**) The E-J characteristics from pre-breakdown region to breakdown region of ZnO piezoresistors prepared with different indium contents. (**B**) The E-J characteristic curves for current densities in the range of 0 to 1.0 × 10^−3^ A/cm^2^.

The nonlinear E-J curve of the zinc oxide varistor from the pre-breakdown region to the breakdown region is shown in Figure 5A. Figure 5B shows the E-J characteristics of the current density from 0 to 1.0 × 10^−3^ A/cm^2^. As shown in Figure 5B, in the pre-breakdown region, the piezosensitive voltage increases rapidly with how the current density is added when the piezoresistor shows a high resistance state, while in the breakdown region, the growth rate of the piezosensitive voltage slows down or even remains unchanged [19].

It can be observed from Figure 5B that when the In^3+^ doping concentration is 0 mol%, the increase in voltage in the corresponding E-J curve starts to change slowly at a current density of 4.0 × 10^−4^ A/cm^2^, and an inflection point appears in the curve trend. Compared with other doping concentrations, the inflection point occurs at the lowest current density of the varistor when the In^3+^ doping concentration is 0.6 mol%, so that the nonlinear coefficient now reaches a maximum value of 62.2 when the In^3+^ doping concentration is 0.6 mol%. This is because the more drastic the change in the inflection point between the pre-breakdown region and the breakdown region is, the greater the nonlinear coefficient will be, and this means that the faster the re-response time will be, the more effective the protection of the device will be [35]. Based on the E-J curve and the above equations, the voltage gradient, leakage current density, and nonlinear coefficient of the ZnO varistor can be calculated, and the results are listed in Table 1.

**Figure 6:**
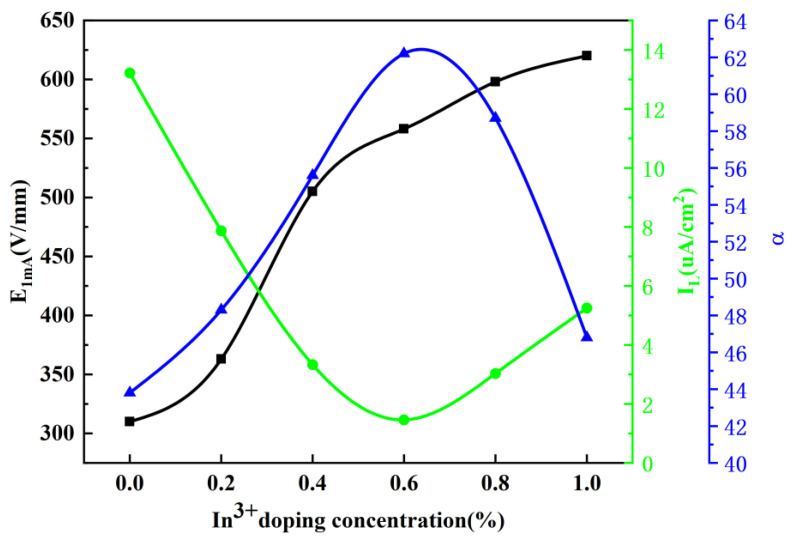
Effect of different In^3+^ doping concentrations on the electrical breakdown strength, nonlinear coefficient, and leakage current of the material.

The effects of different In^3+^ doping concentrations on the electrical breakdown strength, nonlinear coefficient, and leakage current of the materials are shown in Figure 6. The results show that the breakdown voltage increases continuously from 310 V/mm to 620 V/mm with the increase in doping concentration. The increase in breakdown voltage age is partly attributed to the positive effect of Y^3+^ doping. Y^3+^ forms spinel at the grain boundaries during high temperature sintering, which inhibits the growth of the grains and thus reduces the average grain size. The increase in breakdown voltage is partly attributed to the positive effect of Y^3+^ doping. Another reason is that since the ionic radius of In^3+^ is slightly larger than that of Zn^2+^, the solid solution of In^3+^ in ZnO grains has a certain limit. It will not continue to solidify with the increase in In^3+^ doping concentration, and the remaining In^3+^ will remain at the grain boundaries to form a spinel phase, which exists in the triangular region where the grains intersect, restricting the growth of the grains and increasing the voltage gradient. When the doping concentration of In^3+^ is increased from 0.0 mol% to 1.0 mol%, the leakage current (I_L_) decreases rapidly from 13.22 µA/cm^2^ to 1.46 µA/cm^2^, and then rises slowly to 3.32 µA/cm^2^. On the contrary, the nonlinear coefficient (α), which shows a tendency of increasing and then decreasing from 43.8 to 62.8, then decreasing rapidly to 46.8. I_L_ and α showed a negative correlation trend, which is consistent with their [2] findings. The residual voltage ratio (K_p_) value is in the range of 0 to 1.0 mol%. K_p_ values varied between 1.73 and 1.97, with a minimum value of 1.73 for the In^3+^ doping concentration of 0.6 mol%. Thus, improving the protection performance of the arrester. Meanwhile, the different residual pressure ratios provide some evidence that the solid solution of In^3+^ into ZnO grains decreases the grain resistance. The voltage gradient and I_L_ are significantly improved, the α is slightly increased, and the residual voltage ratio is slightly improved compared to the results of Pengfei, M. et al. [36].

**Figure 7:**
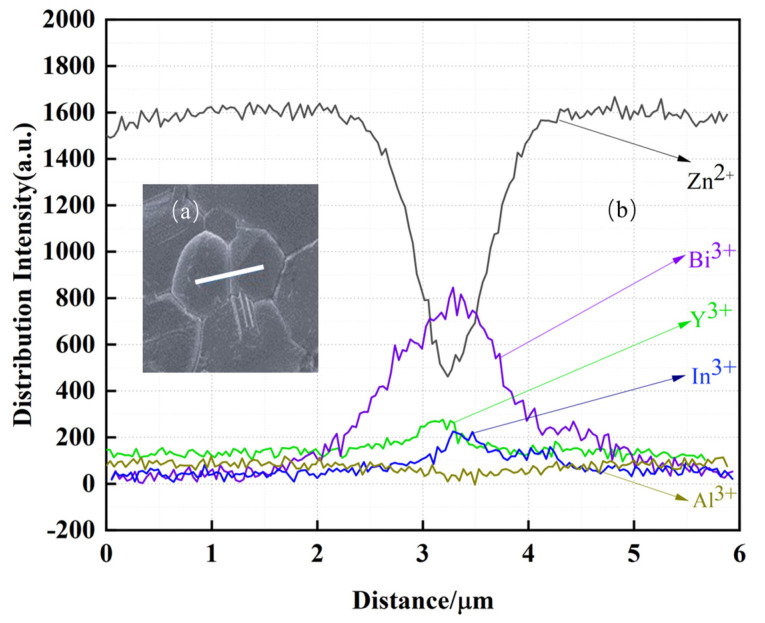
EDX scanning path and element distributions of a typical sample; (**a**) Test Path; (**b**) Intensity of distribution of In^3+^, Al^3+^, and Y^3+^, etc.

Figure 7 shows the EDX analysis image of a typical sample. The scanning image confirms the presence of In^3+^, Al^3+^, and Y^3+^ inside the grains and at the grain edges. During the high temperature sintering process, the dopant ions are solidly dissolved into the defects inside the grains and on the ZnO grain boundaries due to the presence of defects inside the grains and on the ZnO grain boundaries. In Figure 7a, the white line shows the measurement path, which contains two grains and an intergranular layer. Figure 7b shows the intensity of the distribution of In^3+^, Al^3+^, and Y^3+^ on the detecting path. As can be seen in Figure 7b, along the scanning path, the distribution of In^3+^ ions inside the grains is slightly lower than that outside the grains. Some of them are solidly dissolved in the ZnO grains, while others are distributed along the grain boundaries. In Figure 4b, the diffraction angles of the three strongest peaks of the main crystalline phase ZnO are shifted to the lower direction, which also confirms that part of In^3+^ solidifies into the ZnO grains. The Bi element is volatilized in a small amount during the sintering process, and very little solid solution is found inside the grains, which is mainly distributed in the triangular area between the grains.

The distribution intensity of Y^3+^ at grain boundaries is significantly higher than that of Al^3+^ and In^3+^ ions, which can be attributed to two aspects: the higher Y^3+^ doping concentration than that of Al^3+^ and In^3+^ doping concentrations, and the large radius of Y^3+^. The distribution pattern of Al^3+^ ions is opposite to that of Y^3+^ ions because the relationship between the ionic radii of Y^3+^ and Zn^2+^ is: ${R_{{Al}^{2+}}<R}_{{Zn}^{2+}}<R_{Y^{3+}}$. It is easier for Al^3+^ to solidly dissolve into the ZnO grains. Replacing Zn^2+^ with Y^3+^ leads to huge lattice distortions, and for this to happen, more energy needs to be supplied from the outside. This is therefore very difficult to happen, so a large amount of Y^3+^ will accumulate at the grain boundaries.

**Figure 8:**
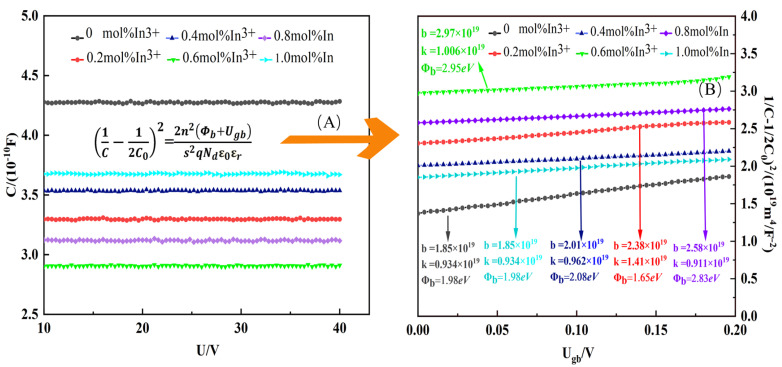
C-V curves of pressure-sensitive ceramics with different In^3+^ doping ratios: (**A**) non-normalized and; (**B**) normalized.

The nonlinear electrical properties are determined by the symmetric double Schottky barriers at the grain boundaries of ZnO. To further investigate the effect of different In^3+^ doping concentrations on the nonlinear characteristics, the capacitance-voltage (C-V) curves of the ZnO varistor are plotted in Figure 8A. It can be seen that the introduction of In^3+^ ions has a first significant effect on the varistor capacitance (C), and the capacitance C value shows a decreasing and then increasing trend as the In^3+^ doping ratio rises. Additionally, no obvious capacitance changes in every sample under the applied bias voltage (U). Based on the following normalized equation [37]:

(2) $${\left(\frac{1}{C}-\frac{1}{{2C}_{0}}\right)}^{2}=\frac{{2n}^{2}\left(\emptyset_{b}+U_{gb}\right)}{s^{2}qN_{d}\varepsilon_{0}\varepsilon_{r}}$$

where *C* and *C*_0_ are the capacitance of the sample with *U_gb_* and zero bias voltage, respectively, *n* is the number of grain boundaries between the electrodes, which can be calculated based on n=h/d (*h* is the thickness of the sample), *U_gb_* is the bias voltage applied to each grain boundary, *s* is the area of the electrodes, *q* is the electronic charge, *ε*_0_ is the vacuum permittivity, and *ε_r_* is the relative permittivity of the ZnO. Based on the normalization equation, the normalization curve is obtained, as shown in Figure 8B. Donor density (*N_d_*) can be calculated from the slope (K) of the straight line and $\emptyset_{b}$ can be calculated from the intercept (b) of the curve. Interface state density (*N_s_*) can be calculated based on ${\emptyset_{b}=qN}_{s}^{2}/2\varepsilon \varepsilon_{0}N_{d}$. The detailed calculation relations are given below [14,37]:

(3) $$b=\frac{n^{2}\emptyset_{b}}{s^{2}qN_{d}\varepsilon_{0}\varepsilon_{r}}$$

(4) $$K=\frac{2n^{2}}{s^{2}qN_{d}\varepsilon_{0}\varepsilon_{r}}$$

By transforming Equations (4) and (5), *N_s_*, *N_d_*, and $\emptyset_{b}$ are calculated as follows:

(5) $$\emptyset_{b}=\frac{b}{k}$$

(6) $$N_{d}=\frac{{2n}^{2}}{s^{2}kq\varepsilon_{0}\varepsilon_{r}}$$

(7) $$N_{s}={\left(\frac{2N_{d}\varepsilon_{0}\varepsilon_{r}\emptyset_{b}}{q}\right)}^{1/2}$$

Parameters such as *N_d_*, *N_s_*, and $\emptyset_{b}$ were calculated based on Figure 8B and the above equation and are listed in Table 1. The interfacial state density *N_s_* of the samples showed an increasing and then decreasing trend with the increase and decrease of In^3+^ doping concentration, which appeared at the peak value of 2.75 × 10^16^ m^−2^ at 0.6 mol% In^3+^ doping. This trend may be caused by the addition of more oxygen vacancies and zinc gaps by the substitution process at the ZnO grain boundaries. *N_d_* and *N_s_* show a similar variational tendency. According to ${\emptyset_{b}=qN}_{i}^{2}/2\varepsilon \varepsilon_{0}N_{d}$, it can be known that ${\;\emptyset }_{b}$ is mainly affected by *N_s_*, and the trend of ${\;\emptyset }_{b}$ tends to be the same as that of *N_s_*. As a result, the value of ${\;\emptyset }_{b}$ increases significantly with In^3+^ ions dopant and reaches up to eV when x = 0.6 mol%, and then decreases to 1.98 eV at x = 1.0 mol% in Table 1.

The radius of the Y^3+^ ion is larger than that of the Zn^2+^ ion, and a small fraction of the Y^3+^ ion is solidly dissolved into the ZnO grains. The ionic radius deviation A value of Y^3+^ ions and Zn^2+^ ions during high temperature sintering of ZnO varistors is 20.4% according to the solid solution ionic formula $\left|(r_{1}-r_{2})/r_{1}\right|$ < A [38] (*r*_1_ denotes ions with large ionic radius values, *r*_2_ denotes ions with small ionic radius values, and A denotes ionic radius deviation). Some of the Y^3+^ ions will act as donors into the ZnO lattice, affecting the donor density *N_d_*. Most of the Y^3+^ ions form fine spinel particles at the edges of ZnO grains, creating a pinning effect that inhibits the growth of ZnO grains [27], increases the number of grain boundaries inside the varistor, and increases the number of Schottky barriers. Significantly improved voltage gradients and nonlinear coefficients.

The radius of Al^3+^ is smaller than that of Zn^2+^, and the absolute value of the difference between the two is larger, so Al^3+^ is easy to solid-solve into ZnO grains, generating a large number of electronic charges, lowering the grain resistance, and keeping the residual pressure ratio low [32,36,39]. At the same time, Al^3+^ doping induces a significant increase in the N_d_ and a slight increase in the surface state density [21], which together lead to a decrease in ${\;\emptyset }_{b}$.

The In^3+^ ionic radius is slightly larger than that of Zn^2+^, and a part of some In^3+^ ions are solidly dissolved into the grains, which acts in the same role as Al^3+^, increasing the electrical conductivity of the ZnO grains, decreasing the grain resistance, and reducing the residual pressure ratio [22]. A fraction of the In^3+^ ions act as donors at grain boundaries, increasing the interface state density (*N_s_*) and enhancing the double Schottky barrier ($\emptyset_{b}$) [2,36]. Furthermore, the residual In^3+^ ions form particles, similar to spinel, that inhibit the growth of ZnO grains. With the further increase of the In^3+^ doping ratio, since the ionic radius of In^3+^ is smaller than that of Y^3+^, it will occupy the position of Y^3+^ in solid solution, which would lead to an excess of Y^3+^ in the grain boundaries. In addition, the liquid phase of Sb-Bi-O appears at high temperatures, which will make the grain spacing larger, leading to the reduction of the Schottky barrier height and the nonlinear coefficient. The grain defect equation for In^3+^-, Al^3+^-, and Y^3+^-doped ZnO can be expressed as [40,41]:

(8) $${Al}_{2}O_{3}\overset{ZnO}{\to }2{Al}_{Zn}^{+}+2e^{-}+2ZnO+1/2O_{2}$$

(9) $${In}_{2}O_{3}\overset{ZnO}{\to }2{In}_{Zn}^{+}+2e^{-}+2ZnO+1/2O_{2}$$

(10) $$Y_{2}O_{3}\overset{ZnO}{\to }2Y_{Zn}^{+}+2e^{-}+2ZnO+1/2O_{2}$$

## 4. Conclusions

In summary, the effects of In^3+^, Y^3+^, and Al^3+^ co-doping on the microscopic and electrical properties of ZnO varistors ceramics were investigated under the precondition of pre-synthesizing BiSbO_4_. The shifts of the three intensity diffraction peaks of ZnO in the XRD tests and the intensity of the distribution of In^3+^ in the grains and grain boundaries in the EDX tests indicate that part of the In^3+^ solid solution is present in the ZnO grains. At the same time, the EDX data show that more Al^3+^ solid solutions are present in the grains, while the opposite is true for Y^3+^, which is mainly related to their ionic radii. In^3+^ and Al^3+^ work together to solid-solve into ZnO grains, replacing Zn^2+^ and providing electrons to keep the residual voltage ratio low. In^3+^ and Y^3+^ work together to stay within grain boundaries and form spinel phases, which are present in the triangular region where the grains meet, limiting the growth of the grains and increasing the voltage gradient. From the C-V test, it is known that some In^3+^ reacts with Zn^2+^ in a defective manner, which leads to an increase in the *N_s_* and the *N_d_* on the grain boundary layer, and the increase of *N_s_* is much larger than that of *N_d_*, so this will lead to an effective increase in the height of the Schottky barrier. The varistor samples doped with 0.6 mol% In(NO_3_)_3_, 0.2 mol% Al(NO_3_)_3_, and 0.9 mol% Y(NO_3_)_3_ show electrical properties characterized by a high voltage gradient, low leakage current, and a low residual voltage ratio. These optimized properties are very useful in the fabrication of metal-oxide surge arresters.

## Figures and Tables

**Table 1:** Microstructure and electrical parameters of varistor samples with various indium contents.

In Content (mol%)	E_1mA_ (V/mm)	J_L_ (µA/cm^2^)	α	d (µm)	N_d_ (10^24^ m^−3^)	N_S_ (10^16^ m^−2^)	$\emptyset_{b}$ (eV)	K_P_
0.0	310	13.22	43.8	6.31	1.63	2.07	0.601	1.97
0.20	360.7	7.87	48.3	6.08	1.72	2.80	1.038	1.82
0.40	505.8	3.33	58.7	5.73	1.94	4.14	2.008	1.76
0.60	558	1.46	62.2	5.23	2.30	5.35	3.03	1.73
0.80	600.5	3.02	55.6	4.77	2.28	5.21	2.83	1.78
1.00	620	3.32	46.8	4.46	2.13	4.30	1.98	1.84

## Data Availability

The data presented in this study are available upon request from the corresponding author. The data are not publicly available due to privacy policies.

## References

[1] Tapan K.G. (1990). Effect of minor doping on the high current application of the ZnO varistor. Ferroelectrics.

[2] Pengfei M., Shanqiang G., Jian W., Jun H., Jinliang H. (2018). Improving electrical properties of multiple dopant ZnO varistor by doping with indium and gallium. Ceram. Int..

[3] Tapan K.G. (1990). Application of Zinc Oxide Varistors. J. Am. Ceram. Soc..

[4] David R.C. (1999). Varistor Ceramics. J. Am. Ceram. Soc..

[5] Jules D.L. (1975). Theory of varistor electronic properties. Crit. Rev. Solid State.

[6] Onreabroy W., Sirikulrat N., Brown A.P., Hammond C.H., Milne S.J. (2006). Properties and Intergranular Phase Analysis of a ZnO–CoO–Bi_2_O_3_ Varistor. Solid State Ion..

[7] Fayat J., Castro M.S. (2003). Defect Profile and Microstructural Development in SnO_2_- Based Varistors. J. Eur. Ceram. Soc..

[8] Ott J., Lorenz A., Harrer M., Preissner E.A., Hesse C., Feltz A., Whitehead A.H., Schreiber M. (2001). The Influence of Bi_2_O_3_ and Sb_2_O_3_ on the Electrical Properties of ZnO-Based Varistors. J. Electroceram.

[9] Liang W., Zhao H., Fan S., Wang H., Zhu Y. (2021). Improvement of voltage gradient and leakage current characteristics of Mn_2_O_3_ and In_2_O_3_ added SnO_2_–ZnO–Ta_2_O_5_ based varistor. Mat. Sci. Semicon Proc..

[10] Li S., Mirlekar G., Ruiz-Mercado G.J., Lima F.V. (2016). Development of chemical process design and control for sustainability. Processes.

[11] Stucki F., Greuter F. (1990). Key role of oxygen at zinc oxide varistor grain boundaries. Appl. Phys. Lett..

[12] Ramírez M.A., Sim Es A.Z., Bueno P.R., Márquez M.A., Orlandi M.O., Varela J.A. (2006). Importance of oxygen atmosphere to recover the ZnO-based varistors properties. J. Mater. Sci..

[13] Bueno P.R., Leite E.R., Oliveira M.M., Orlandi M.O., Longo E. (2001). Role of oxygen at the grain boundary of metal oxide varistors: A potential barrier formation mechanism. Appl. Phys. Lett..

[14] Lin W., Xu Z., Wang Z., Yang J., Chu R. (2020). Influence of Bi_3_Zn_2_Sb_3_O_14_ pre-synthesis phase on electrical properties of the ZnO-Bi_2_O_3_ based varistor ceramics. J. Alloy Compd..

[15] Leng S., Li G., Zheng L., Cheng L., Zeng J. (2011). Influences of Ba/Ti Ratios on the Positive Temperature Coefficient of Resistivity Effect of Y-Doped BaTiO_3_–(Bi_1/2_Na_1/2_)TiO_3_ Ceramics. J. Am. Ceram. Soc..

[16] Wenjin L., Jianpin A., Xiaoxuan S. (2019). Effect of Presynthesis of Bi_2_O_3_ and Sb_2_O_3_ on Microstructure and Electrical Properties of High Performance ZnO-Bi_2_O_3_-based Varistor Ceramics. J. Ceram..

[17] Imai T., Udagawa T., Ando H., Tanno Y., Kayano Y., Kan M. (1998). Development of high gradient zinc oxide nonlinear resistors and their application to surge arresters. IEEE Trans. Power Deliv..

[18] He J.L., Hu J., Lin Y.H. (2009). The high voltage gradient ZnO varistorswith rare earths doped. Sci. China Ser. E Technol. Sci..

[19] Roy S., Das D., Roy T.K. (2018). Influence of sintering temperature on microstructure and electrical properties of Er_2_O_3_ added ZnO-V_2_O_5_-MnO_2_-Nb_2_O_5_ varistor ceramics. J. Alloys Compd..

[20] Houabes M., Bernik S., Talhi C., Bui A. (2005). The effect of aluminium oxide on the residual voltage of ZnO varistors. Ceram. Int..

[21] Wangcheng L., Jun H., Jun L., Jinliang H., Rong Z. (2010). The Effect of Aluminum on Electrical Properties of ZnO Varistors. J. Am. Ceram. Soc..

[22] Pengfei M., Jun H., Hongfeng Z., Jinliang H. (2016). High voltage gradient and low residual-voltage ZnO varistor ceramics tailored by doping with In_2_O_3_ and Al_2_O_3_. Ceram. Int..

[23] Hongfeng Z., Jun H., Shuiming C., Qingyun X., Jinliang H. (2016). Improving age stability and energy absorption capabilities of ZnO varistors ceramics. Ceram. Int..

[24] Nahm C.W. (2011). Varistor properties of ZnO-Pr_6_O_11_-CoO-Cr_2_O_3_-Y_2_O_3_-In_2_O_3_ ceramics. Met. Powder Rep..

[25] Bueno P.R., Varela J.A., Longo E. (2008). SnO_2_, ZnO and related polycrystalline compound semiconductors: An overview and review on the voltage-dependent resistance (non-ohmic) feature. J. Eur. Ceram. Soc..

[26] Ashraf M.A., Bhuiyan A.H., Hakim M.A., Hossain M.T. (2011). Microstructure and electrical properties of Sm_2_O_3_ doped Bi_2_O_3_-based ZnO varistor ceramics. Mater. Sci. Eng. B.

[27] Nahm W.C. (2014). Microstructure and varistor properties of Y_2_O_3_-doped ZnO–Pr_6_O_11_–CoO–Cr_2_O_3_–La_2_O_3_ ceramics. Ceram. Int..

[28] Shahraki M.M., Mahmoudi P., Abdollahi M., Ebadzadeh T. (2018). Fine-grained SnO_2_ varistors prepared by microwave sintering for ultra-high voltage applications. Mater. Lett..

[29] Bernik S., Maček S., Bui A. (2004). The characteristics of ZnO–Bi_2_O_3_-based varistor ceramics doped with Y_2_O_3_ and varying amounts of Sb_2_O_3_. J. Eur. Ceram. Soc..

[30] Wurst J.C., Nelson J.A. (2010). Lineal Intercept Technique for Measuring Grain Size in Two-Phase Polycrystalline Ceramics. J. Am. Ceram. Soc..

[31] Sasikumar K., Bharathikannan R., Raja M., Mohanbabu B. (2020). Fabrication and characterization of rare earth (Ce, Gd, and Y) doped ZrO_2_ based metal-insulator-semiconductor (MIS) type Schottky barrier diodes. Superlattice Microst.

[32] Raship N.A., Tawil S.N.M., Nayan N., Ismail K. (2023). Effect of Al Concentration on Structural, Optical and Electrical Properties of (Gd, Al) Co-Doped ZnO and Its n-ZnO/p-Si (1 0 0) Heterojunction Structures Prepared via Co-Sputtering Method. Materials.

[33] Das S., Das S., Das D., Sutradhar S. (2017). Tailoring of room temperature ferromagnetism and electrical properties in ZnO by Co (3d) and Gd (4f) element co-doping. J. Alloy Compd..

[34] Yusoff M.M., Mamat M.H., Ismail A.S., Malek M.F., Khusaimi Z., Suriani A.B., Mohamed A., Ahmad M.K., Rusop M. (2018). Enhancing the performance of self-powered ultraviolet photosensor using rapid aqueous chemical-grown aluminum-doped titanium oxide nanorod arrays as electron transport layer. Thin Solid. Film..

[35] Liu W., Zhang L., Kong F., Wu K., Li J. (2020). Enhanced voltage gradient and energy absorption capability in ZnO varistor ceramics by using nano-sized ZnO powders. J. Alloy Compd..

[36] Pengfei M., Jun H., Jinliang H. (2017). Low-residual-voltage ZnO varistor ceramics improved by multiple doping with gallium and indium. Mater. Lett..

[37] Cui F., Xu Z., Chu R., Li G. (2020). Improving electrical properties of ZnO–Bi_2_O_3_–Sb_2_O_3_–MnO_2_ varistors by doping with pre-synthesized Bi–Si–O phase. J. Alloy Compd..

[38] Liu D.J., Ma Y.Y., He J.B., Wang H., Zhou Y.X., Sun G.Y., Zhao H.F. (2023). ZnO varistors with low leakage current and high stability arrester with Ga doping. Acta Phys. Sin..

[39] Anand V., Sakthivelu A., Kumar K.D.A., Valanarasu S., Ganesh V., Shkir M., Kathalingam A., AlFaify S. (2018). Novel rare earth Gd and Al co-doped ZnO thin films prepared by nebulizer spray method for optoelectronic applications. Superlattices Microstruct..

[40] Bernik S., Mac Ek S.O., Ai B. (2001). Microstructural and Electrical Characteristics of Y_2_O_3_-Doped ZnO–Bi_2_O_3_–Based Varistor Ceramics. J. Eur. Ceram. Soc..

[41] Tietze T., Audehm P., Chen Y.C., Schütz G., Straumal B.B., Protasova S.G., Mazilkin A.A., Straumal P.B., Prokscha T., Luetkens H. (2015). Correction: Corrigendum: Interfacial dominated ferromagnetism in nanograined ZnO: A μSR and DFT study. Sci. Rep..

